# Exploring eco-anxiety in Italian adolescents: psychometric evaluation of the Climate Change Anxiety Scale and theoretical insights into the association with pro-environmental attitudes

**DOI:** 10.3389/fpsyg.2025.1601891

**Published:** 2025-11-05

**Authors:** Matteo Innocenti, Sara Bocci Benucci, Giulia Dockerty, Giulio De Micco, Gaia Surya Lombardi, Alessio Perilli, Giulia Congedo, Mattia Di Russo, Stefania Bruno, Giulia Fioravanti

**Affiliations:** ^1^Department of Life Sciences and Public Health, Università Cattolica del Sacro Cuore, Rome, Italy; ^2^Department of Experimental and Clinical Medicine, University of Florence, Florence, Italy; ^3^Department of Health Sciences, University of Florence, Florence, Italy

**Keywords:** climate change anxiety, eco-anxiety, adolescents, climate change worry, pro-environmental attitudes

## Abstract

**Introduction:**

Climate change significantly impacts the health and future of adolescents, yet they have limited ability to prevent its effects, leaving them especially vulnerable to climate anxiety. The present study aims to first explore the psychometric properties of the Climate Change Anxiety Scale among adolescents in Italy (Study 1), and to investigate the psychological pathways through which climate change anxiety impacts adolescents’ pro-environmental attitudes, examining the mediating roles of climate change worry and rumination related to eco-anxiety (Study 2).

**Methods:**

In Study 1, the psychometric properties (i.e., dimensionality, internal consistency, sex invariance and convergent validity) of the CCAS were explored using a sample of 250 high school students (45.60% *F*, *M*_age_ = 16.13, SD_age_ = 1.44). In Study 2, the mediation model was tested in a new sample of 250 high school students (51.60% *F*, *M*_age_ = 16.12, SD_age_ = 1.58).

**Results:**

In Study 1, the CCAS showed a two-factor structure (i.e., cognitive impairment and functional impairment) with a good fit [*χ*^2^ (df) = 83.980(64), *p* = 0.05; RMSEA [90% CI] = 0.02[0.002;0.025]; CFI = 0.995; SRMR = 0.054]. McDonald’s Omega values were 0.91 and 0.87. Sex invariance was obtained only at the configural level. Both the CCAS factors were positively correlated with climate change worry, whereas only cognitive impairment was positively associated with pro-environmental attitudes. In Study 2, results of the mediation model showed that higher CCAS predicted both higher climate change worry and higher rumination related to eco-anxiety, which in turn predicted higher pro-environmental attitudes. The direct path from CCAS to pro-environmental attitudes was also significant, indicating a negative relationship. The model explained 17% of the total variance, and all the indirect effects were significant.

**Discussion:**

The CCAS showed satisfactory psychometric properties among Italian adolescents. The exploratory model suggests that in adolescents, worry and rumination may have an adaptive role by transforming climate change anxiety into pro-environmental attitudes.

## Introduction

The advancing climate crisis is unfolding in a way that goes beyond environmental degradation. The multifaceted nature of this crisis includes a variety of impacts on human health, which are both physical and psychological. In particular, a growing body of research reveals the climate crisis’s potential to act as a “risk multiplier,” exposing vulnerable populations to increased risks due to climate change and pre-existing social, economic, and health inequalities (Lawrance et al., 2022).

While the physical health consequences of climate change due to rising temperatures, extreme weather events (EWEs), resource scarcity, and increased disease transmission are increasingly well-documented (Haines et al., 2020; Tong et al., 2022; Whitmee et al., 2015), the psychological ramifications, especially for young people, demand further investigation.

The subjective experience of climate change can be complex and nuanced, manifesting in emotions such as anxiety, fear, anger, despair, pain, and a sense of powerlessness (Clayton and Karazsia, 2020; McQueen, 2021; Ojala et al., 2021; Stanley et al., 2021). It is becoming increasingly clear that these emotions are a rational response to the threats posed by climate change, not a pathological condition (Verplanken et al., 2020). The tangible risks of the climate crisis to human health and the continuous degradation of ecological systems make it difficult to distinguish between pathological and physiological responses. Climatic phenomena provoke emotional reactions that can have a significant impact on psychological wellbeing, often resulting in cognitive distortions and alterations in behavioral patterns, such as not being capable of enacting pro-environmental behaviors. Within the context of climate change mitigation and adaptation, as well as in response to other natural disasters, worry can be understood as a normative and adaptive psychological process that facilitates preparedness for potential threats. However, when such worry is excessively driven by anxiety, it becomes overwhelming and difficult to regulate (Barlow, 2002; Reser, 2004). This anxiety and the more persistent concern, often referred to as “climate change worry,” can manifest in diverse ways, ranging from heightened emotional distress to profound feelings of hopelessness and powerlessness (Boluda-Verdu et al., 2022). Research has shown that individuals experiencing frequent and severe climate worry often report significant impairments in daily functioning, affecting social relationships, work performance, and overall wellbeing (Lenhard et al., 2024). Notably, a substantial proportion of those affected also exhibit symptoms of depression and sleep disturbances, further exacerbating their psychological burden.

The American Psychological Association (APA) has formally recognized the adverse effects of climate change on mental health, citing increased rates of stress, depression, and anxiety (American Psychological Association, 2017). Research consistently shows that climate change perception and awareness are strongly associated with mental health outcomes, including depression, anxiety, stress, and even suicidal ideation (Gianfredi et al., 2024; Cianconi et al., 2020; Whitmarsh et al., 2022).

The elements that can trigger a negative emotional response are environmental disasters, direct exposure to EWEs, the growing awareness of environmental degradation and future threats, and the perceived insufficiency of current attempts to mitigate the effects of climate change (Pihkala, 2020; Gianfredi et al., 2024). Studies suggest that individuals with a heightened perception of climate change report lower levels of wellbeing and resilience, with consequences on daily functioning, including disruptions in sleep, appetite, and cognitive performance (Gianfredi et al., 2024). Moreover, climate anxiety—characterized by persistent worry, obsessive thinking, and a sense of helplessness—has emerged as a growing concern. This phenomenon, also referred to as “eco-anxiety” or “climate change anxiety,” has been linked to increased rates of adjustment disorders, substance use, and emotional distress, further underscoring the profound psychological toll of the climate crisis (Gianfredi et al., 2024).

### Adolescents and climate change anxiety

Modern social structures are characterized by widespread access to a large amount of climate-related information, which often emphasizes catastrophic outcomes. These scenarios frequently exacerbate feelings of vulnerability and uncertainty, especially among adolescents who are in the process of development and identity formation (Burgess et al., 2022). A global survey has revealed the extent of widespread distress among children and young population. In 2021, Hickman and colleagues conducted a global survey of 10,000 young people (aged 16–25) in 10 countries, which found extensive climate anxiety and significant negative impacts on daily functioning. The majority reported at least moderate concern, experiencing a range of negative emotions and feeling betrayed by a perceived lack of competence in government responses. Eco-anxiety among adolescents interacts with other societal uncertainties regarding medium-term futures, such as employment or housing (Atkinson, 2024). It also intersects with generational tensions tied to the politics of self-management, where some adults interpret adolescent anxiety as either a sign of insufficient resilience or, more harshly, as generational narcissism—despite there being no evidence to support such views (Arnett, 2013). One of the key elements described by adolescents in relation to experiences of eco-anxiety comes from the extent to which the adult population and governments appear not to care (Hickman et al., 2021), and place the responsibility on adolescents to take action. Positioning the responsibility for action on the individual can intensify rather than alleviate eco-anxiety (Atkinson, 2024). Adolescents will face the inevitable climate change challenge in the future, but no effective tools or strategies have been provided to face this threat (Hurley et al., 2022).

Given these evidences, it is crucial to assess the nature and extent of climate-change anxiety among adolescents, the degree to which such anxiety and worries interfere with daily functioning, and the perceived level of control adolescents have over the climate-related worry process, to develop targeted interventions that enhance mental wellbeing and promote adaptive coping mechanisms.

To achieve these aims, adequate measurement instruments for assessing climate change anxiety among adolescents are needed. In this regard, the Climate Change Anxiety Scale (CCAS; Clayton and Karazsia, 2020) is considered a reliable and valid tool for assessing climate change anxiety. In particular, the CCAS focuses specifically on cognitive and functional impairment related to anxiety caused by climate change (Clayton and Karazsia, 2020). Cognitive impairment refers to difficulty sleeping or concentrating, and nightmares or crying in response to climate change; functional impairment reflects the interference of climate change concerns with a person’s ability to work or socialize. The CCAS was originally developed in samples of adults (i.e., aged 18 and above) and has been validated in several countries, including Italy. However, the Italian validation (Innocenti et al., 2021) has also been conducted in an adult population, not in adolescent samples. Since climate change anxiety primarily affects adolescents, accurately measuring the construct requires determining whether the scale developed and validated for adults also adequately captures this construct in younger populations. This would allow for a more accurate investigation of climate change anxiety in relation to other variables, thereby helping to identify key areas for preventive and therapeutic interventions. Therefore, the primary objective of the current study (Study 1) is to investigate the psychometric properties of the CCAS among a sample of Italian adolescents. Given the favourable properties of the CCAS among adults and the applicability of its item contents among adolescents, studying its psychometric properties in adolescents could help determine its appropriateness in youth.

This study also extends the literature concerning the validity of the scale by exploring measurement invariance across sex. Currently, there is no evidence about the ability of the CCAS to measure climate change anxiety equivalently across sexes in adolescents. Measurement invariance is necessary to determine whether the scores between groups are comparable and have the same meaning across the groups (Reise et al., 1993). This is a relevant issue for the literature on climate change anxiety in adolescents, since employing invariant instruments will also allow us to investigate the effect of biological sex more fairly on climate change anxiety among adolescents.

The second aim of the current study (Study 2) is to investigate the psychological pathways through which climate change anxiety and eco-anxiety impact adolescents’ pro-environmental attitudes, examining the mediating roles of climate change worry and rumination related to eco-anxiety. The rationale for this model is grounded in recent literature (e.g., Boluda-Verdu et al., 2022), showing that worry represents the cognitive component of climate change anxiety and can operate as a double-edged process. On the one hand, moderate levels of worry may facilitate adaptive problem solving, preparedness, and engagement in pro-environmental behaviors; on the other hand, excessive worry may reinforce maladaptive rumination, amplify distress, and impair daily functioning (Orrù et al., 2024). From this perspective, climate change worry and rumination may mediate the impact of climate change anxiety on adolescents’ pro-environmental attitudes.

## Study 1

The purpose of Study 1 is to explore the psychometric properties (i.e., dimensionality, internal consistency, sex invariance and convergent validity) of the CCAS in a sample of Italian adolescents.

## Materials and methods

### Participants and procedures

A total of 250 Italian adolescents (45.60% females, *M*_age_ = 16.13, SD_age_ = 1.44) attending high school in Italy were recruited using convenience and snowball sampling methods, provided they met the following inclusion criteria: aged between 14 and 18 years, of Italian nationality, and residing in Italy. Exclusion criteria included illiteracy or inability to provide consent or to complete the survey online. Participants were recruited through social network announcements using a convenience sampling approach, and recruitment took place from March to August 2024. The rule of thumb, which is to have at least 10 participants for each item (Costello and Osborne, 2005), was followed during the recruitment procedure.

All participants were informed that their participation was voluntary, anonymous and confidential. A web link directed the participants to the study website. The first page of the online survey explained the study’s general purpose. Those who declared they were at least 14 years old and who consented to take part in the study were redirected to the second page of the survey, which contained questions about socio-demographic information (i.e., gender and age), two questions concerning climate crisis (i.e., “How informed do you consider yourself to be on the topic of climate change?”; “Have you had direct experience with events caused by climate change?”). Then, participants were asked to respond to four self-report questionnaires. Participants did not receive any compensation, and the study procedures were conducted in accordance with the Declaration of Helsinki. The Institutional Review Board of the University of Florence approved the study (Protocol number: 0114884).

### Measures

#### Climate Change Anxiety Scale

The Italian version (Innocenti et al., 2021) of the 13-item Climate Change Anxiety Scale (CCAS; Clayton and Karazsia, 2020) was administered. The scale assesses self-perceived anxiety about climate change. The Italian version of the scale presents a bifactorial structure addressing cognitive and functional impairment. Participants are requested to respond on a 5-point Likert scale ranging from 1 (*Never*) to 5 (*Almost always*). Higher scores indicate higher climate change anxiety. A sample item is “Thinking about climate change makes it difficult for me to concentrate” for the cognitive impairment factor and “My concerns about climate change make it hard for me to have fun with my family or friends” for the functional impairment factor. The Italian version showed good psychometric properties among Italian adults (Innocenti et al., 2021).

#### Climate Change Worry Scale

The Italian version (Innocenti et al., 2022) of the 10-item Climate Change Worry Scale (CCWS; Stewart, 2021) was used to measure self-perceived worry about climate change. Participants were asked to answer on a 5-point Likert scale ranging from 1 (*Never*) to 5 (*Always*), and higher scores indicate higher worry related to climate change. A sample item is “I worry about climate change more than others.” The CCWS has been previously validated on Italian adolescents, showing good psychometric properties (Donati et al., 2024). In the current sample, Cronbach’s alpha was 0.93, and McDonald’s omega was 0.93.

#### New Ecological Paradigm Scale-Revised

The Italian version (Prati et al., 2011) of the 15-item New Ecological Paradigm Scale-Revised (NEP-R; Dunlap et al., 2002) was used to measure personal attitudes, beliefs and values about environmental protection. Items are presented on a 4-point Likert scale ranging from 1 (*Strongly disagree*) to 4 (*Strongly agree*). The scale presents two factors: the *dominant social paradigm* (NEP-DSP) and the new social p*aradigm* (NEP-NSP). A sample item for the NEP-DSP is “Humans have the right to modify the natural environment to suit their needs”; for the NEP-NSP it is “We are approaching the limit of the number of people the earth can support.” Agreement with NEP-NSP items and disagreement with the NEP-DSP items indicate pro-environmental orientations (pro-NEP responses). In the present study, we created an overall environmental attitudes score by reversing the negatively worded items (i.e., the NEP-DSP subscale), ensuring that higher scores consistently indicated stronger pro-environmental attitudes. This scoring procedure has been implemented in previous research with Italian samples (e.g., Prati et al., 2015). In our current sample, the scale demonstrated acceptable internal consistency with Cronbach’s alpha of 0.72 and McDonald’s omega of 0.76.

### Statistical analyses

Multivariate normality was assessed using Mardia’s test (Mardia, 1970), which indicated a violation of multivariate normality in terms of skewness (b1d = 159.99, *χ*^2^(455) = 6666.60, *p* < 0.001) and kurtosis (b2d = 518.96, *z* = 129.69, *p* < 0.001) therefore, Confirmatory Factor Analyses (CFA) with Weighted Least Squares Mean and Variance adjusted (WLSMV) estimation method was conducted to verify the factor structure previously identified in the Italian version of the CCAS among adults (Innocenti et al., 2021). The CFA was performed using R software’s Lavaan package (Rosseel, 2012). Standard goodness-of-fit indices were selected *a priori* to assess the measurement models (Hu and Bentler, 1999): the *χ*^2^ (and its degrees of freedom and *p*-value), the Standardized Root Mean Square Residual (SRMR—Jöreskog and Söbom, 1993) “close to” 0.09 or lower, the Comparative Fit Index (CFI—Bentler, 1995) “close to” 0.90 or higher (Hu and Bentler, 1999)., and the Root Mean Square Error of Approximation (RMSEA—Steiger, 1990) less than 0.08 (Browne and Cudeck, 1992). Next, internal consistency was calculated using Cronbach’s alpha, McDonald’s Omega, and item-total correlations.

Measurement invariance by sex was calculated using a multi-group CFA. Hierarchically nested models were applied to test configural, metric, and scalar invariance. Configural invariance refers to whether the same CFA is valid in each group; metric invariance concerns the equivalence of the factorial loadings across groups; and scalar invariance is assumed when the item intercepts and the factor loadings are equally constrained across groups. The criteria for assessing differences between competing models were based on multiple indicators: (1) the scaled difference chi-square test (Satorra and Bentler, 2010), (2) the difference in CFIs between nested models (Cheung and Rensvold, 2002), (3) the difference in RMSEA values, and (4) the difference in SRMR values (Chen, 2007). When ΔCFI between two nested models is greater than 0.01, it is assumed that the additional constraints have led to a poorer fit and the more constrained model is rejected; for ΔRMSEA, a difference of less than 0.015 between models suggests that the more constrained model fits the data equally well or better, and can be retained and for ΔSRMR, a difference of less than 0.03 indicates that the additional constraints have not significantly worsened the model fit, and the more constrained model can be retained.

Finally, convergent validity was evaluated by calculating Pearson’s correlations between the CCAS, the CCWS and the NEP-R scores.

## Results

The majority of the sample (57.2%) reported being quite informed about climate change, followed by 30.4% who considered themselves little informed, 8.8% who reported being better informed than average, 2% who answered “very informed,” and a small percentage of participants (1.6%) who answered “not informed at all.” In response to the question about direct experiences with events caused by climate change (such as floods, landslides, or geological issues), 65.6% of respondents stated they had never experienced such events. However, 22.4% reported having had such experiences once, and 10.8% had faced these events more than once. Only 1.2% of participants reported experiencing these events frequently.

### Confirmatory factor analysis

**Figure 1:**
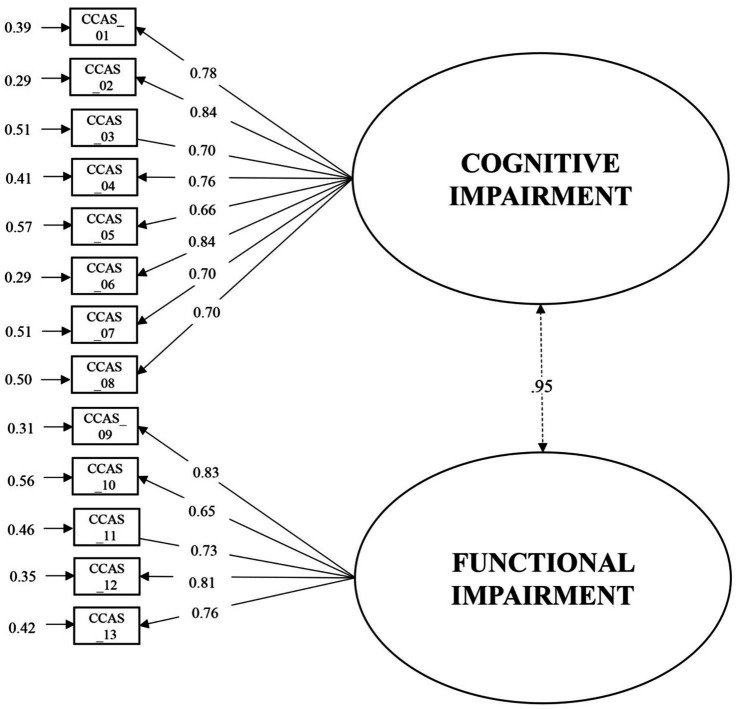
Results of the confirmatory factor analysis for the CCAS.

The factor structure of the CCAS was tested with a Confirmatory Factor Analysis. The CCAS showed a two-factor structure with a good fit [*χ*^2^ (df) = 83.980(64), *p* = 0.05; RMSEA [90% CI] = 0.02[0.002; 0.025]; CFI = 0.995; SRMR = 0.054]. Factor loadings for all items on the two factors were good, with each standardized loading exceeding 0.60 (Tabachnick and Fidell, 2013; See Figure 1).

### Internal consistency

Cronbach’s alpha values were 0.91 for the cognitive impairment factor and 0.87 for the functional impairment factor. McDonald’s Omega values were 0.91 for the cognitive impairment factor and 0.87 for the functional impairment factor. Descriptive statistics for each item of the CCAS and item-total correlations for each item within its respective subscale are reported in Table 1.

**Table 1:** Mean, standard deviation, and item-total correlation of the CCAS.

Cognitive impairment factor	Mean ± SD	Item-total correlation	Functional impairment factor	Mean ± SD	Item-total correlation
CCAS_01	1.65 ± 0.99	0.74	CCAS_09	1.25 ± 0.71	0.84
CCAS_02	1.30 ± 0.78	0.80	CCAS_10	1.40 ± 0.87	0.57
CCAS_03	1.27 ± 0.76	0.65	CCAS_11	1.30 ± 0.81	0.75
CCAS_04	1.21 ± 0.66	0.76	CCAS_12	1.38 ± 0.88	0.73
CCAS_05	1.97 ± 1.05	0.63	CCAS_13	1.34 ± 0.83	0.66
CCAS_06	1.40 ± 0.83	0.81			
CCAS_07	1.28 ± 0.75	0.68			
CCAS_08	1.50 ± 0.85	0.63			

### Sex invariance

A multigroup CFA analysis was conducted to evaluate measurement invariance across boys and girls. Model fit indices were examined, including the model chi-square value, CFI, RMSEA, and SRMR. Given the chi-square statistic’s high sensitivity to sample size (Bentler and Bonett, 1980), we determined measurement invariance across groups by comparing all model fit indices, following established recommendations for acceptable change criteria. The fit indices of the model split by gender (configural invariance) seemed acceptable: *χ*^2^ = 134.083, df = 128, *p* = 0.34; *χ*^2^ /df = 1.04; RMSEA [90%CI] = 0.02 [0.00–0.049]; CFI = 0.97; SRMR = 0.07. However, when testing for metric invariance, the fit indices deteriorated substantially, with a ΔCFI of −0.051 and a ΔRMSEA of 0.017, both exceeding the recommended thresholds of 0.01 and 0.015, respectively (Cheung and Rensvold, 2002; Chen, 2007). The change in SRMR (ΔSRMR = 0.041) also exceeded the suggested cutoff of 0.03 (Chen, 2007). These results indicate that imposing equal factor loadings across groups led to a poorer fit, suggesting that full metric invariance was not supported.

Subsequently, the comparison between the scalar and metric models showed minimal changes in fit indices (ΔCFI = −0.009, ΔRMSEA = 0.005, ΔSRMR = 0.003), suggesting that the scalar constraints did not further deteriorate the fit. However, since the metric model presented unacceptable fit indices, the results indicated that full scalar invariance could not be retained without further compromising the model’s adequacy.

In conclusion, while the changes between the metric and scalar models were minimal, the overall fit remained inadequate, notably after introducing metric constraints. The results support configural invariance, meaning that the basic factor structure is equivalent across sex, even though the strength of the relationships (metric invariance) and the intercepts (scalar invariance) differed significantly between boys and girls. Thus, the basic dimensional structure holds across groups, but more refined comparisons (i.e., factor loadings and intercepts) should be interpreted cautiously when comparing scores between sexes.

### Convergent validity

Descriptive statistics and Pearson’s correlations are shown in Table 2. Both the CCAS factors showed positive correlations with climate change worry, whereas cognitive impairment was positively associated with pro-environmental attitudes.

**Table 2:** Descriptive statistics and correlations among the CCAS and the other variables assessed.

	*M* ± SD	1.	2.	3.	4.
1. CCAS—cognitive impairment	11.57 ± 5.23	–	0.84^**^	0.74^**^	0.20^**^
2. CCAS—functional impairment	6.66 ± 3.33		–	0.65^**^	0.12
3. CCWS	21.35 ± 8.91			–	0.42^**^
4. NEP-R	42.86 ± 5.69				–

**p < 0.001; CCAS, Climate Change Anxiety Scale; CCWS, Climate Change Worry Scale; NEP-R, New Ecological Paradigm-revised.

## Study 2

The purpose of Study 2 is to investigate the psychological pathways through which climate change anxiety impacts adolescents’ pro-environmental attitudes, examining the mediating roles of climate change worry and rumination related to eco-anxiety.

## Materials and methods

### Participants and procedures

A new sample of 250 Italian adolescents (51.60% female, *M*_age_ = 16.12, SD_age_ = 1.58) attending high schools in Italy was recruited. The school offices of three Italian regions (i.e., Piedmont, Lazio, and Campania) were contacted and received a letter presenting the project and requesting the schools’ participation. The letter specified that the project was financed by the Italian Ministry of University and Research (MUR) under the projects PRIN 2022—Projects of National Relevance (project code: 2022N22J5F, CUP: B53D2302054000) and that participants would not receive incentives or benefits for their participation. The same inclusion criteria as those in Study 1 were adopted, and the study procedure was identical to that of Study 1. The recruitment was conducted between January and April 2025. All informed consents were collected from students and their parents. The Institutional Review Board of the University of *** approved the study (Protocol number: 0274167).

### Measures

#### Hogg Eco-Anxiety Scale

The Italian version (Rocchi et al., 2023) of the 13-item Hogg Eco-Anxiety Scale (HEAS; Hogg et al., 2021) was administered to measure eco-anxiety symptoms in the past 2 weeks. The Italian scale version presents a four-factor structure addressing affective symptoms, rumination, behavioral symptoms and anxiety about personal impact. Items are presented on a 4-point Likert scale ranging from 0 (*not at all*) to 3 (*nearly every day*), asking about the frequency of the symptoms of eco-anxiety experienced in the past 2 weeks. A sample item is “[in the past 2 weeks, I felt] Unable to stop thinking about future climate change and other global environmental problems.” The Italian version showed good psychometric properties among Italian adults (Rocchi et al., 2023) and adolescents (Spano et al., 2025) In the current sample, Cronbach’s alpha values were 0.79 for the affective symptoms factor, 0.74 for the rumination factor, 0.72 for the behavioral symptoms factor and 0.85 for the anxiety about personal impact factor. McDonald’s Omega values were 0.79, 0.77, 0.74, 0.85, respectively.

#### Climate Change Anxiety Scale

The Italian version (Innocenti et al., 2021) of the 13-item Climate Change Anxiety Scale (CCAS; Clayton and Karazsia, 2020) was administered to measure self-perceived anxiety about climate change. A detailed description of the scale is provided in Study 1. In the current sample, Cronbach’s alphas were 0.85 for the cognitive impairment factor and 0.84 for the functional impairment factor. McDonald’s omega was 0.85 for both the cognitive and functional impairment factors.

#### Climate Change Worry Scale

The Italian version (Innocenti et al., 2022) of the 10-item Climate Change Worry Scale (CCWS; Stewart, 2021) was employed to assess self-perceived worry regarding climate change. A detailed description of the scale can be found in Study 1. In the current sample, Cronbach’s alpha was 0.90, and McDonald’s omega was 0.90.

#### New Ecological Paradigm Scale-Revised

The Italian version (Prati et al., 2011) of the 15-item New Ecological Paradigm Scale-Revised (NEP-R; Dunlap et al., 2002) was used to measure personal attitudes, beliefs and values about environmental protection. A detailed description of the scale is provided in Study 1. In the current sample, Cronbach’s alpha was 0.64, and McDonald’s omega was 0.67.

### Statistical analyses

All the analyses were performed using IBM Statistical Package for the Social Sciences (SPSS), version 29.0 (IBM Corp., Armonk, NY, USA). Pearson’s correlations between the CCAS, the CCWS, the HEAS and the NEP-R scores were first calculated. Then, to test the model, a parallel mediation model using the PROCESS macro for SPSS (model 4), developed by Hayes (2013), was estimated. In this model, the CCAS served as the predictor, the CCWS and the subscale rumination of the HEAS were the mediators, and the NEP was the criterion variable. Additionally, gender was added as a covariate in the model. Bias-corrected bootstrap confidence intervals (CIs) derived from 5,000 bootstrap resamples were estimated to test for the significance of conditional direct and indirect effects. The effects were considered significant if the CI values did not include zero.

## Results

The majority of the sample (58.8%) declared to consider themselves quite informed about climate change, followed by 27.60% who considered themselves little informed, 8.4% who reported being better informed than average, 2.4% who answered “very informed” and a low percentage of participants (2.8%) who answered “not informed at all.” In response to the question about direct experiences with events caused by climate change (such as floods, landslides, or geological issues), 57.20% of respondents stated they had never experienced such events. However, 27.2% reported having had such experiences once, and 13.2% had faced these events more than once. Only 2.4% of participants reported experiencing these events frequently.

### Correlations

Descriptive statistics and Pearson’s correlations are shown in Table 3. The CCAS showed a positive correlation with climate change worry and the ruminative dimension of the HEAS. Both worry and rumination were positively associated with pro-environmental attitudes. The CCAS was not associated with pro-environmental attitudes.

**Table 3:** Descriptive statistics and correlations among the study variables.

	*M* ± SD	1.	2.	3.	4.	5.	6.	7.	8.	9.
1. HEAS-13—affective symptoms	2 ± 2.25	–	0.65^**^	0.40^**^	0.59^**^	0.51^**^	0.41^**^	0.51**	0.45^**^	0.18^**^
2. HEAS-13—rumination	1.39 ± 1.62		–	0.33^**^	0.64^**^	0.53^**^	0.44^**^	0.53^**^	0.53^**^	0.27^**^
3. HEAS-13—behavioral symptoms	1.03 ± 1.77			–	0.29^**^	0.49^**^	0.48^**^	0.53^**^	0.31^**^	0.02
4. HEAS-13—anxiety about personal impact	1.80 ± 2.00				–	0.44^**^	0.33^**^	0.43^**^	0.58^**^	0.38^**^
5. CCAS—cognitive impariment	11.49 ± 4.41					–	0.73^**^	0.95^**^	0.62^**^	0.10
6. CCAS—functional impariment	6.69 ± 3.03						–	0.90^**^	0.57^**^	−0.03
7. CCAS—total score	18.15 ± 6.92							–	0.64^**^	0.06
8. CCWS	20.12 ± 7.70								–	0.30^**^
9. NEP	43.24 ± 4.71									–

**p < 0.001; CCAS, Climate Change Anxiety Scale; CCWS, Climate Change Worry Scale; HEAS-13, Hogg Eco-Anxiety Scale; NEP = New Ecological Paradigm.

### Mediation model

**Figure 2:**
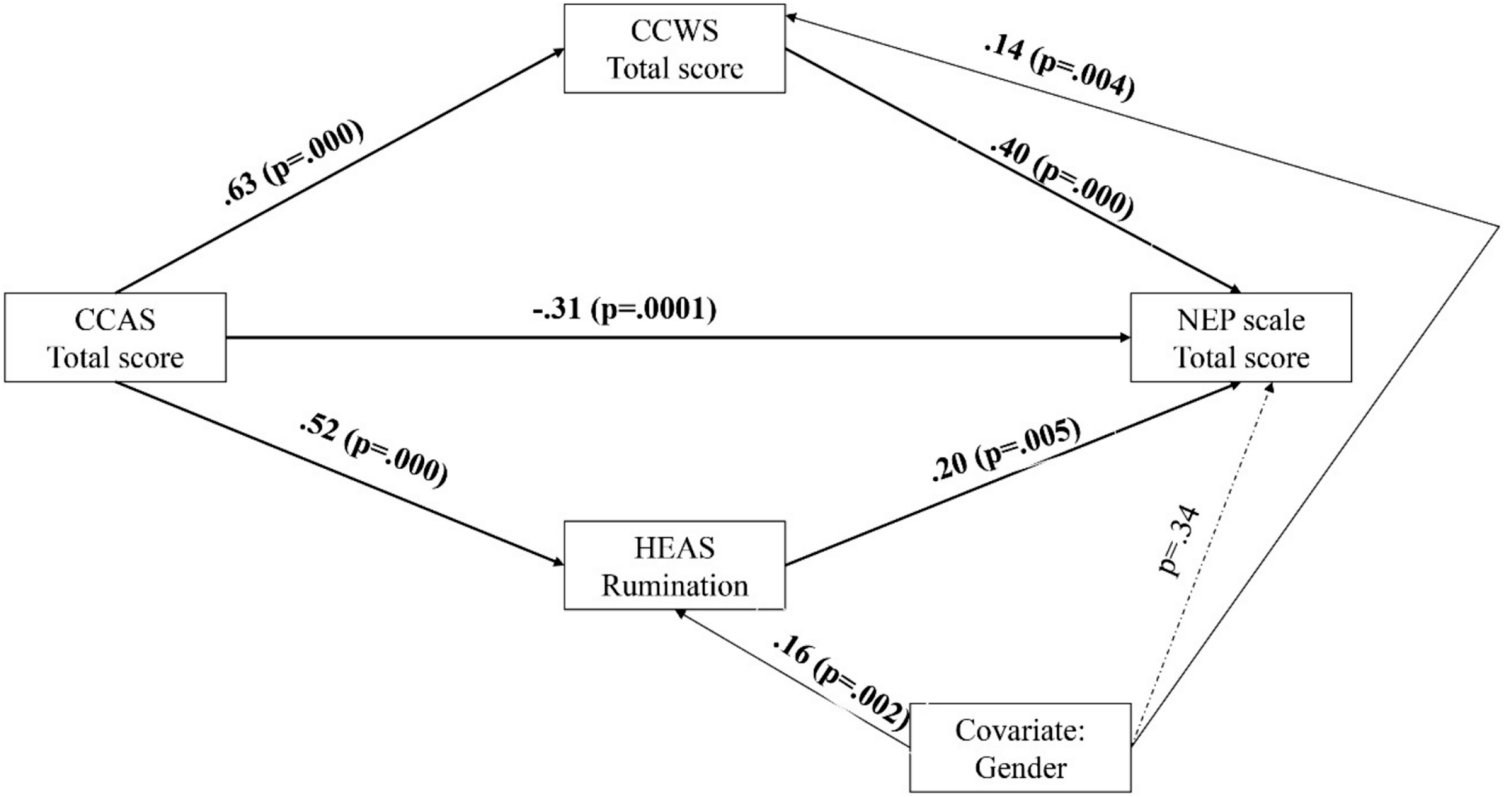
Results of the model tested.

The results of the model tested are shown in Figure 2; all coefficients are standardized. As displayed, higher CCAS predicted both higher CCWS and higher rumination assessed with the HEAS, which in turn predicted higher pro-environmental attitudes. The direct path from CCAS to pro-environmental attitudes was also significant, indicating a negative relationship: higher climate change anxiety was associated with lower pro-environmental attitudes. All indirect effects were significant (total indirect effect = 0.24; 95% CI: [0.16, 0.33]; indirect effect of CCWS = 0.25; 95% CI: [0.13, 0.38]; indirect effect of HEAS rumination = 0.11; 95% CI: [0.02, 0.19]). In addition, gender (female) predicted rumination and climate change worry. The model accounted for 17% of the total variance.

## Discussion

The recent increase in the prevalence of emotions such as eco-anxiety and climate distress presents the scientific community with the urgent need to develop solid and reliable psychometric tools to assess anxiety induced by direct or indirect exposure to climate change and environmental degradation. This need is even more crucial for adolescents, who have a unique perspective on the future and are in a critical stage of development, during which they learn to manage and regulate their emotions. These characteristics make individuals in this age group particularly vulnerable to the emotional burden triggered by environmental concerns. This situation, combined with the fact that scale such as the CCAS has demonstrated excellent psychometric properties in adults, highlights the importance of assessing its psychometric properties within specific age groups and cultural contexts to ensure its suitability across different applications.

Therefore, the first aim of the current study was to explore the psychometric properties (i.e., dimensionality, internal consistency, sex invariance and convergent validity) of the CCAS in a sample of Italian adolescents.

Study 1 results showed that the Italian version of the CCAS demonstrates satisfactory psychometric properties (i.e., internal consistency and validity) among adolescents, in line with previous studies conducted among Italian adults (Innocenti et al., 2021). The factor structure is consistent with that reported in the original version (Clayton and Karazsia, 2020), as well as in other studies from different countries (e.g., Mouguiama-Daouda et al., 2022). However, we did not find support for full sex invariance of the CCAS among adolescents. This result is inconsistent with a previous study that found the original two-factor CCAS to be equivalent across men and women (Larionow et al., 2022). However, this finding aligns with Hogg et al. (2023), who found that only configural invariance was demonstrated across sexes, whereas metric and scalar invariance were not obtained. Further studies are needed to test the CCAS invariance across sex groups. Establishing full measurement invariance would ensure that any observed score differences between boys and girls accurately represent true differences in climate change anxiety levels rather than measurement artefacts.

In addition to the validation findings, the exploratory model tested in Study 2 offers further insights into the processes underlying climate change anxiety in adolescents.

Specifically, our results suggest that climate change worry and eco-anxiety rumination mediate the relationship between climate change anxiety and pro-environmental attitudes. This finding is consistent with previous evidence indicating that worry constitutes the cognitive dimension of anxiety and can play both adaptive and maladaptive roles depending on its intensity and regulation (Orrù et al., 2024). Our results highlight that in adolescents, worry and rumination may help transform climate change anxiety into preparedness and pro-environmental attitudes. Indeed, climate change anxiety was found to have a negative direct impact on pro-environmental attitudes, suggesting that when climate change concerns interfere with cognitive functioning and with a person’s ability to work or socialize, it can lead to emotional distress and feelings of helplessness. It can be difficult to focus on day-to-day responsibilities, maintain productivity, or engage in meaningful pro-environmental activities (Lenhard et al., 2024). This is alarming because individuals may fall into maladaptive coping strategies, such as eco-paralysis, leading to feelings of overwhelm and demotivation (Innocenti et al., 2023; Sampaio et al., 2023). However, when climate change anxiety prompts worry and rumination, this can enhance pro-environmental attitudes, values, and beliefs. These results align with theoretical perspectives that conceptualize eco-anxiety as an adaptive emotional response capable of motivating individuals to engage in more pro-environmental behaviors (Pihkala, 2020).

Taken together, the current results suggest that the complex interplay between eco-anxiety, related cognitive strategies (i.e., worry and rumination) and pro-environmental attitudes calls for multi-faceted intervention strategies that not only address anxiety management but also empower adolescents to engage in pro-environmental behaviors.

This study should be interpreted in light of some limitations. First, while the analyses of the psychometric properties of the CCAS provide valuable insights, the recruited sample may not fully represent the entire Italian adolescent population. Although the sample was deemed adequate for psychometric testing, large-scale studies are necessary to improve the reliability and generalizability of the findings. Secondly, the use of self-reported measures may introduce response biases, as participants may either underestimate or overestimate their experiences of eco-anxiety or climate change anxiety and related concerns. Additionally, another limitation is the cross-sectional design of the study, which prevents drawing causal conclusions about the relationships between variables. From this perspective, the mediation model should be interpreted with caution. Future research employing larger adolescent samples and longitudinal designs will be essential to replicate and extend these findings, as well as to refine the role of worry and rumination in shaping the impact of eco-anxiety on environmental attitudes and behaviors and to understand the evolution of climate change-related dynamics and eco-anxiety over time.

Mapping the trajectory of these experiences could facilitate the development of targeted interventions to promote resilience and adaptive coping strategies, helping young people navigate an uncertain future. Finally, although the study provides significant insights within the Italian context, its local focus may limit the external validity of the findings. Cultural, social, and economic factors influencing adolescent experiences of eco-anxiety may vary across countries, making cross-cultural validation essential to broaden the applicability of the results. Consequently, future research should include diverse samples from different cultural contexts to substantiate these findings and develop universally relevant interventions to address eco-anxiety and climate change anxiety among adolescents.

In spite of the limitations mentioned above, the current findings have some practical implications. Given the current and future situation, caregivers and mental health practitioners will increasingly need to consider stressors such as climate change anxiety when working with adolescents, in both the diagnostic phase and therapeutic interventions. Since an increasing number of young people are concerned about the planet and its future, integrating climate-related psychological support techniques into counselling and support services will become increasingly crucial. This could play a central role in mitigating anxiety, teaching emotional regulation strategies to promote resilience, and fostering adaptation.

Encouraging open dialogue—both between caregivers and young people and among peers—about fears and concerns related to climate change can enhance adolescents’ ability to process their emotions and shift toward active engagement through pro-environmental behaviors rather than feeling powerless and at risk of eco-paralysis. Indeed, the literature extensively documents that engaging in pro-environmental behaviors not only generates an energizing experience that motivates individuals to act in response to a perceived threat but also, in turn, helps reduce levels of eco-anxiety (Barrett and Russell, 1999; Innocenti et al., 2023; Sangervo et al., 2022; Stanley et al., 2021; Heeren et al., 2022; Sampaio et al., 2023).

## Conclusion

Given that the psychological effects of climate change, as well as concerns about it, are primarily increasing among adolescents, the results of this research address the growing need to have useful tools to accurately assess climate change among youth. Nonetheless, testing a theoretical model on the psychological processes involved, in addition to the validation study, allows the psychometric results to be embedded in a broader conceptual framework and highlights potential mechanisms through which eco-anxiety exerts its effects in younger populations. Although the mediation analysis should be regarded as exploratory, the present findings lay the groundwork for future research aimed at understanding, preventing, and addressing the psychological consequences of the climate crisis in younger generations.

## Data Availability

The raw data supporting the conclusions of this article will be made available by the authors, without undue reservation.
